# Ultramarathon and Renal Function: Does Exercise-Induced Acute Kidney Injury Really Exist in Common Conditions?

**DOI:** 10.3389/fspor.2019.00071

**Published:** 2020-01-21

**Authors:** Mathias Poussel, Charlie Touzé, Edem Allado, Luc Frimat, Oriane Hily, Nathalie Thilly, Hélène Rousseau, Jean-Charles Vauthier, Bruno Chenuel

**Affiliations:** ^1^Center of Sports Medicine and Adapted Physical Activity, University Hospital of Nancy, Vandoeuvre-lès-Nancy, France; ^2^EA 3450 DevAH-Development, Adaptation and Disadvantage, Cardiorespiratory Regulations and Motor Control, Université de Lorraine, Nancy, France; ^3^Department of General Practice, Maison de Santé des Trois Monts, Dommartin-les-Remiremont, France; ^4^Nephrology Department, University Hospital of Nancy, Vandoeuvre-lès-Nancy, France; ^5^Department of Methodology, Promotion and Investigation in Clinical Research, University Hospital of Nancy, Vandoeuvre-lès-Nancy, France

**Keywords:** exercise physiology, extreme endurance, acute renal injury, performance, biomarker

## Abstract

**Background:** Increasing ultramarathons participation, investigation into strenuous exercise and kidney function has to be clarified.

**Study Design:** Prospective observational study.

**Methods and Protocol:** The study used data collected among ultra-marathon runners completing the 2017 edition of the 120 km “Infernal trail” race. Samples were collected within 2 h pre-race (start) and immediately post-race (finish). Measurements of serum creatinine (sCr), cystatin C (Cys), creatine kinase, and urine albumin were completed. Acute Kidney Injury (AKI) as defined by the RIFLE criteria. “Risk” of injury was defined as increased serum Creatinine (sCr) × 1.5 or Glomerular Filtration Rate (GFR) decrease >25%. Injury was defined as 2 × sCr or GFR decrease >50%. These two categories of AKI were combined to calculate total incidence at the finish line. GFR was estimated by two methods, using measure of sCr and using measure of cystatin C. Urinary biomarkers [neutrophil gelatinase-associated lipocalin (NGAL)] were also used to define AKI. Outcome results before and after the race were compared by using McNemar test for qualitative data and Wilcoxon signed-rank test for quantitative data, in modified intent-to-treat and per-protocol analyses.

**Results:** A sample of 24 included finishers, with no use of non-steroidal anti-inflammatory drugs (NSAIDs) was studied. Depending the methodology used to calculate GFR, the prevalence of AKI was observed from 0 to 12.5%. Urinary biomarkers of kidney damage were increased following the race but with no significant decrease in GFR.

**Discussion/Conclusion:** Our study showed a very low prevalence of AKI and no evidence that ultra-endurance running can cause important kidney damage in properly hydrated subjects with no use of NSAIDs. Whether the increase in urinary biomarkers of kidney damage following the race reflects structural kidney injury or a simple metabolic adaptation to strenuous exercise needs to be clarified.

## Introduction

Ultramarathons, consisting of running and walking, often on mountain trails, over a distance longer than the classical marathon (Zaryski and Smith, 2005), have become more and more popular over the past decade (U.S. Road Race Trends, 2018).

Under extreme conditions, both exerted by environments and/or exercise, physiology is pushed to its limits and the risk of exercise-induced injuries is high (Millet and Millet, 2012; Knechtle and Nikolaidis, 2018). Among them, acute kidney injury, involving altered renal function, has often been reported in sports with prolonged and strenuous exercise, such as marathon running and has also been documented in ultramarathons (Boulter et al., 2011; Knechtle and Nikolaidis, 2018). Lipman et al. (2016, 2017) have found a prevalence of an acute kidney injury in ultra-marathon running close to 50% of all runners. A peculiar context associating dehydration and/or use of non-steroidal anti-inflammatory drugs (NSAIDs) have been documented in order to promote acute kidney injury associated to ultra-marathons (Hodgson et al., 2017; Lipman et al., 2017; Knechtle and Nikolaidis, 2018). Therefore, whether ultra-marathon running by itself increases the risk of acute kidney injury need to be clarified. Some recent studies have shown an unchanged glomerular filtration rate by ultramarathon (Wołyniec et al., 2018), or no evidence that prior acute kidney injury caused greater renal dysfunction from a subsequent exercise stimulus (Hoffman and Weiss, 2016). We wanted to test the hypothesis that in common conditions (no use of NSAIDs and no major risk of dehydration), ultra-distance race has no supplemental short-term impact on acute kidney function.

Then, in order to identify the real deleterious consequences of ultramarathon running on kidney function, we used a very strict protocol and assessed the glomerular filtration rate and urinary biomarkers among a sample of runners with no recent use of non-steroidal anti-inflammatory drugs, before and following a 120 km ultramarathon (with 5,700 m of positive height gain). Our hypothesis was that in common conditions, ultra-distance running by itself does not necessarily provide a greater risk for AKI.

## Methods and Protocol

The “Infernal-trail” race −10 and 11th of September 2017:

This race is the closest ultramarathon race to our University Hospital and we take part of the medical coverage of this sporting event. This race is referenced by the International Trail Running Association (five points assigned for the 120 km race). We studied a sample of volunteers among the 224 participants of a famous French ultra-trail across the Vosges mountains, entitled “Infernal-trail,” during its 2017 edition. The main characteristics of this race were: 120 km trail-run, 5,700 m of positive elevation changes. The temperature during the run rose from 8.6°C (6 h) to 11.1°C (14–16 h), on a rainy day (8 mm of rain over 24 h) with relative humidity from 89 to 99% and maximal wind speed of 46.7 km/h. The maximum time allowed for race completion was 30 h and the race started at 6 a.m. on the 10th of September 2017.

During the race the organizer provided 8 stations offering food and beverages, such as hypotonic sports drinks, tea, soup, caffeinated drinks, water, fruit, such as bananas, chocolate, energy bars, and bread. These food and drinks stations were readily accessible along the route.

The study was a prospective observational study, approved by the French Ethics Committee of the North-Western part of France (“Comité de Protection des Personnes Nord-Ouest IV”), under the reference n° 2017-A00808-45. The study was registered in Clinical Trials under the reference NCT 03136315.

### Subjects

The previous month and the day before the race, all participants received information via e-mail about the study, its procedure, benefits and risks associated. Participation in the study was suggested but was voluntary and not compensated. If a participant has agreed to participate, he (she) signed a written informed consent outlining study requirements, at race registration the morning before the run. Eligible participants were older than 20 years, with no chronic health disease and no drug treatment, and consent to participate. More specifically, exclusion criteria included ingestion of non-steroidal or corticoid anti-inflammatory drugs the week before the race. All included participants completed a self-administered questionnaire on subject characteristics, including anthropometric data, daily physical activity and ultramarathon-running experience (racing and training). They also confirmed the lack of use of any medication the week before the race. They underwent blood and urine samples within 2 h before the start of the run, and again immediately after finishing the run (<20 min after the end for each runner). This maximal delay of 20 min following the end of the race has been chosen in respect to kinetics of blood and urinary biomarkers following the completion of exercise, in order to increase homogeneity of biological samples and avoid any pre-analytics bias. More particularly, previous studies have shown that the peak increase in urinary neutrophil gelatinase-associated lipocalin (uNGAL) is reached immediately and 25 min following a high intensity exercise (McCullough et al., 2011; Junglee et al., 2012). Venous blood samples were drawn from the antecubital vein in a sitting position and collected in a SST gel separator tube (BD Vacutainer). Serum was separated by centrifuging samples at 1,000 G for 10 min and frozen at −80°C until analysis. Urine collection was performed before and after the run. Urine samples were also stored frozen at −80°C until analysis.

### Study Outcomes

The primary outcome was the prevalence of Acute Kidney Injury (AKI), assessed by the established RIFLE criteria for severity of AKI using either parameter: decrease in Glomerular Filtration Rate (GFR) or increase in serum creatinine (sCr) (Bellomo et al., 2004; Venkataraman and Kellum, 2007). “Risk” of injury was defined as increased sCr × 1.5 or GFR decrease >25%. Injury was defined as 2 × sCr or GFR decrease >50%.

GFR was estimated by two methods:
- Using measure of serum cystatin C (Cys) and GFR CKD-EPI cystatin C equation from the Chronic Kidney Disease—Epidemiology Collaboration (Inker et al., 2012).
  $$\begin{array}{l} GFR\;CKD-EPI\;cystatin\;C\;\left(ml/min/1.73m^{2}\right)=133\\ \;\times {min\left(\frac{Cys}{0.8}\right)}^{-0.499}\times {\max\left(\frac{Cys}{0.8},\;1\right)}^{-1.328}\\ \;\times {\;0.996}^{Age}\;\left[\times 0.932\;if\;Female\right]\; \end{array}$$
  where Cyst is serum cystatin C, min is the lowest value between Cyst/0.8 and 1, max is the highest value between Cyst/0.8 and 1.
- Using measure of sCr and GFR CKD-EPI creatinine equation (Levey et al., 2009) in order to allow comparisons with previous studies.

In both cases we used the term “eGFR” to highlight the fact the GFR was estimated (eGFR CKD-EPI cystatin C and eGFR CKD-EPI creatinine).

The second outcome was a potential renal damage following the race, reflected by increased urinary levels of damage-associated nephron biomarker, neutrophil gelatinase-associated lipocalin (NGAL) (Paragas et al., 2011; Singer et al., 2011; Nickolas et al., 2012).

Since urinary markers are able to change considerably over time, urinary parameters were standardized basing on the ratio uNGAL/uCr, in order to normalize the effect of strenuous exercise on diuresis and used its changes from the start to the end of the race.

$$\begin{array}{l} change\;uNGAL/uCr\;\left(\%\right)=\frac{\left(uNGAL/uCr\;finish\right)-\left(uNGAL/uCr\;start\right)}{\left(uNGAL/uCr\;start\right)}\\ \;\times 100\; \end{array}$$

We applied the Reference Change Value (RCV) method to identify significant changes in uNGAL/uCr with $RCV=Z\times \sqrt{2}\times \sqrt{CVa^{2}+CVi^{2}}$ (Fraser, 2011) with *Z* = 1.65 for *p* < 0.05, CVi, intra-individual coefficient of variability = 88% from Delanaye et al. (2011) and Cva (analytical coefficient of variability) was determined by the laboratory, from internal quality control techniques, repeating the measure on the same urinary sample.

Samples were analyzed at the Hospital “Emile Durkheim” laboratory in Epinal, France, which has accreditation from the French Centre of Accreditation. Serum cystatin C was measured by the Roche cystatin C assay on the Cobas® 6000 c501 analyser (CVa = 9.6%). uNGAL was measured using the ELISA method (Human NGAL ELISA kit 036, Bioporto Diagnostics®) performed manually. Serum and urinary concentration of creatinine were measured using the Roche enzymatic method on the Cobas® 6000 c501 (CVa = 1.7–1.0%, respectively).

### Statistical Analysis

Subject characteristics and outcome parameters were described by numbers and percentages (and CI 95% for outcomes) for qualitative data or by medians and maximum/minimum for quantitative data. Outcome results before and after the race were compared by using McNemar test for qualitative data and Wilcoxon signed-rank test for quantitative data.

Modified intent-to-treat analysis (mITT) was conducted on all included participants who underwent blood and urine samples before the start and after the end of the race. Per-protocol analysis was conducted on participants considered in mITT and with no protocol deviation.

The analyses were performed using SAS version 9.4 (SAS Institute, Inc., Cary, N.C.), a threshold of *p* = 0.05 for two-tailed tests being considered significant.

## Results

**Figure 1:**
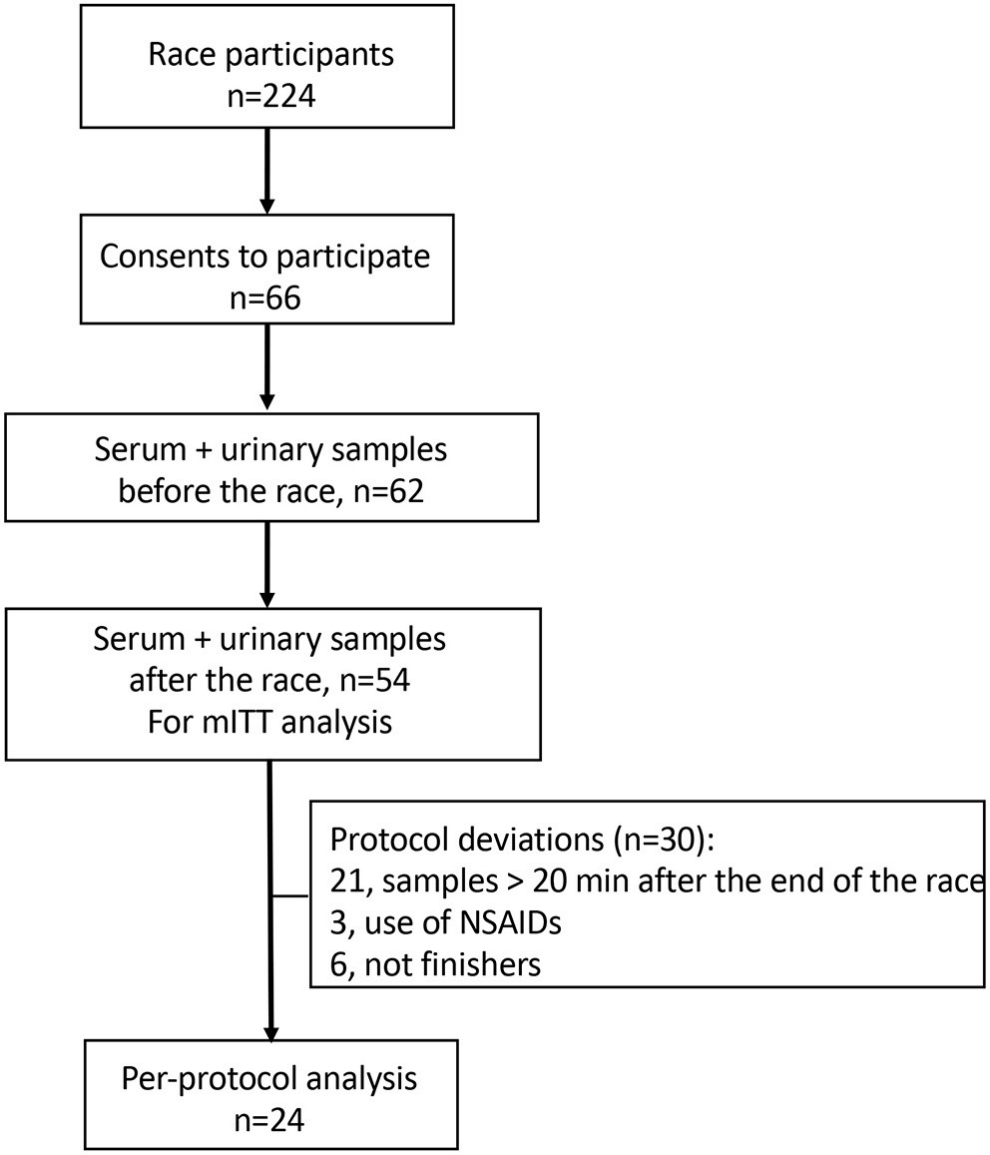
The study flow chart. mITT analysis, modified intent-to-treat analysis; NSAIDs, non-steroidal anti-inflammatory drugs.

A total of 66 subjects were enrolled in the study, among 224 participants of the race (participation rate = 29.5%). Twelve volunteers missed the data collection before the start or following the end of the race. Of the 54 participants undergoing testing and considered in the mITT analysis, 30 were excluded from the per-protocol analysis (Figure 1). General characteristics of participants considered in mITT and in per-protocol analyses are detailed in Table 1 showing that both groups were quite similar.

**Table 1:** Participant individual and race characteristics.

	**Subjects included in mITT** **analysis**					**Subjects included in per-protocol** **analysis**				
	***N***	**%**	**Median**	**Min**	**Max**	***N***	**%**	**Median**	**Min**	**Max**
Age (years)	54		38	24	68	24		36.5	24	57
**Gender**										
Male	53	98.1				23	95.8			
Female	1	1.9				1	4.2			
Body weight (kg)	54		72	55	88	24		71.5	55	87
Body height (cm)	54		176	163	192	24		177	166	189
Body mass index (kg/m^2^)	54		22.9	19.4	26.5	24		22.6	19.5	26.5
Practice of ultramarathons (years)	54		4	0	32	24		4	1	32
Prior completed ultramarathons (number)	54		6	0	20	24		7	2	20
Number of completed ultramarathons on 3 last years	54		1	0	10	24		1.5	0	10
Average training week time^*^ (h/week)	54		8	3	18	24		8	3	15
Average training week running distance^*^ (km/week)	54		60	10	150	24		70	30	150
**Race outcome**										
Finisher	48	88.9				24	100			
Withdrawal	6	11.1				0	0			
Finish time (h)	54		23.9	9	29.9	24		23.1	16,6	29.9
Average speed (km/h)	54		4.9	4	7.2	24		5.2	4.0	7.2

* *Average training week running distance and time among last 3 months before the race (km/week and h/week)*.

*min, minimum; max, maximum*.

### Estimated Glomerular Filtration Rate

eGFR CKD-EPI cystatin C increased significantly from the beginning to the end of the race about 4.5 ml/min/1.73 m^2^ (*p* = 0.04). However, the difference in eGFR CKD-EPI creatinine prior and after the race was not statistically significant (*p* = 0.08), and in average decreased by 3.5 ml/min/1.73 m^2^, as described in Table 2.

**Table 2:** Changes of serum biomarkers of acute kidney injury, serum cystatin C, and serum creatinine.

	***N***	**Median**	**Min**	**Max**
Pre-race serum cystatin C (mg/l)	24	0.8	0.5	1.1
Post-race serum cystatin C (mg/l)	24	0.8	0.5	1
Pre-race eGFR CKD-EPI cystatin (ml/min/1.73 m^2^)	24	113.5	74	147
Post-race eGFR CKD-EPI cystatin C (ml/min/1.73 m^2^)	24	118.5	83	148
Δ eGFR CKD-EPI cystatin C (post-race–pre-race) (ml/min/1.73 m^2^)	24	4.5	−23	39
Variation rate GFR CKD-EPI cystatin C (%)	24	3.8	−15.6	52.7
Pre-race serum creatinine (mg/l)	24	8.6	6.4	12
Post-race serum creatinine (mg/l)	24	8.9	6.6	13.7
Pre-race eGFR CKD-EPI creatinine (ml/min/1.73 m^2^)	24	107	75	133
Post-race eGFR CKD-EPI creatinine (ml/min/1.73 m^2^)	24	102	69	135
Δ eGFR CKD-EPI creatinine (post-race–pre-race) (ml/min/1.73 m^2^)	24	−3.5	−39	28
Variation rate eGFR CKD-EPI creatinine (%)	24	-3.3	-36.1	32.9

*CKD-EPI, Chronic Kidney Disease-Epidemiology Collaboration; eGFR, Estimated Glomerular Filtration Rate; Δ, difference between level post-race–level pre-race; N, number; min, minimum; max, maximum*.

### Prevalence of Acute Kidney Injury (AKI)

Following the race and compared to pre-race level, we observed decrease in eGFR DFG CKD-EPI cystatin C in 7 subjects (29.2%). No subject presented any acute kidney injury or risk of injury, according to the RIFLE classification.

Nevertheless, we found decrease in eGFR CKD-EPI creatinine in 16 subjects (66.7%). The overall prevalence of risk of AKI at the end of the race was at 1/24 (4.2%). No subject presented acute renal *failure*, nor *Injury* level. Using the KDIGO classification (Kidney Disease Improving Global Outcome) (Kellum et al., 2013), currently recommended (≥1.5 × baseline serum creatinine and/or serum creatinine ≥26.5 μmol/l), the prevalence of acute kidney injury was still 1/24 (4.2%).

### Biomarkers of Inflammation and Potential Renal Damage

The variation rate of the ratio *uNGAL*/*uCr* was found greater than the reference change value in 3 subjects (12.5%, *p* < 0.001; confidence interval 95% [2.8–33.6]) (Table 3).

**Table 3:** Urinary biomarkers of renal damage: urinary neutrophil gelatinase-associated lipocalin (NGAL) and urinary creatinine (*N* = 24).

	**Median**	**Min**	**Max**
Pre-race uNGAL (ng/ml)	20.1	2	328.8
Post-race uNGAL (ng/ml)	53.1	2	376.1
Δ uNGAL (post–pre-race) (ng/ml)	10.2	−273.2	351.9
Pre-race uCr (mg/ml)	0.9	0.1	2.7
Post-race uCr (mg/ml)	1.4	0.4	2.6
Δ uCr (post–pre-race) (mg/ml)	0.5	−1.7	2
Pre-race uNGAL/uCr (ng/mg)	23.7	1.5	517.8
Post-race uNGAL/uCr (ng/mg)	35.5	0.9	313.4
Δ uNGAL/uCr (post–pre-race) (ng/mg)	−2.3	−475.7	308.8

*min, minimum; max, maximum; N, number; uCr, urinary concentration of creatinine; uNGAL, urinary concentration of neutrophil gelatinase-associated lipocalin; Δ, difference between level post-race–level pre-race*.

## Discussion

This study is the first, to the authors' knowledge, that uses several ways to precisely assess kidney function following a real ultra-marathon race of 120 Km, among a sample of finishers and using a very strict protocol in order to avoid pre-analytics bias (maximal delay for the biological sample following the end of the race <20 min principally) and the confounding influence of the use of NSAIDs (criterion of exclusion).

Then, the prevalence of acute kidney injury was found at 0% and the risk of AKI at 4.2% using classical methods of GFR calculations and RIFLE or KDIGO criteria in order to define AKI, to 12.5% using urinary biomarkers for AKI.

We showed that eGFR CKD-EPI cystatin C significantly increased from the start to the end of the race (*p* = 0.04) while the eGFR CKD-EPI creatinine did not change significantly, with tendency of a decrease (*p* = 0.08). Therefore, an ultra-marathon race in common conditions and without any use of NSAIDs induced no systematic and prolonged drop in renal perfusion, sufficient to disrupt the physiological mechanisms enable to maintain GFR during exercise.

Using eGFR CKD-EPI creatinine calculation to define AKI we observed an over-estimation of its prevalence compared to eGFR CKD-EPI cystatin C method. It was already demonstrated that a proportion of the sCr rise observed on extreme exercise reflects increase in muscle disruption, rather than a decrease in GFR (Irving et al., 1990; Hodgson et al., 2017). Since cystatin C is less affected by muscle mass and diet than creatinine is, it has been anticipated that estimation of GFR by cystatin C would be more accurate than would by creatinine (Vinge et al., 1999; Stevens et al., 2009; Tangri et al., 2011; Inker et al., 2012). Indeed, studies on kidney function in runners following a marathon demonstrated that the mean cystatin C was half that of the mean sCr rise (Mingels et al., 2009). Then, as strenuous exercise can lead to severe muscle damage with rhabdomyolysis, the use of serum creatine could be greatly modified and misleading for the GFR estimation. A recent review has recommended that the use of sCr to estimate GFR under conditions of extreme exercise should be avoided (Hodgson et al., 2017).

In previous studies, a greater prevalence of AKI has been found in marathoners, from 33 to 84% (McCullough et al., 2011; Hewing et al., 2015; Mansour et al., 2017), apart from the fact that these results were obtained by eGFR CKD-EPI creatinine method. Some major differences were found between marathoners and ultra-marathoners: former run much slower in training than marathoners but complete more running kilometers and more running hours per weeks (Rüst et al., 2012; Knechtle and Nikolaidis, 2018). Moreover, it has been demonstrated that short and fast ultra-marathons are more likely to cause kidney injury than longer ultra-marathons at a lower speed (Shin et al., 2016). Then speed appeared to be crucial in the pathophysiology of temporary reduction in renal function due to strenuous exercise, inducing a greater decrease in GFR at high speed.

Some risk factors for kidney damage in ultra-marathons have been identified as female sex, low body weight and a significant weight loss during the run (Lipman et al., 2016). Since our sample included only one female ultra-marathoner, then we could have underestimated the prevalence of AKI in a general population of runners.

Further risk factors for acute kidney injury in ultra-endurance runners have been demonstrated as the use of NSAIDs (Lipman et al., 2017) sometimes leading to dramatic clinical situation of acute renal failure needing hospitalization (Poussel et al., 2013). In a previous “Infernal-Trail” race (2014), we observed a prevalence of self-medication during the race at 18% and NSAIDs has been consumed in half of the cases (Didier et al., 2017). It has been reported that up to 75% of ultra-endurance athletes use NSAIDs during the race (Wharam et al., 2006). Our present results, excluding subjects with the use of NSAIDs demonstrated a high decreased prevalence of AKI compared to studies whose included them (McCullough et al., 2011; Hewing et al., 2015; Mansour et al., 2017), highlighting the deleterious effects on renal function of the use of NSAIDs during an ultramarathon race, able to exacerbate renal injury (Lipman et al., 2017).

Our results have demonstrated that levels of NGAL significantly increased during the race. NGAL is a glycoprotein released during early phases of a post-ischemic kidney in response to kidney injury, inflammation, and oxidative stress (Mishra et al., 2005; Ronco, 2007). It has been considered as a new biomarker for renal disease and particularly important in the early detection of an AKI (Ronco, 2007; Lippi et al., 2012). More specifically, NGAL is produced by the kidney proximal tubules and the increase in levels of NGAL following the race may indicate tubular injury independently of any change of in GFR in our study. Whether these results reflect a physiological adaptation through metabolic adaptation to exercise or a pathological kidney damage needs to be clarified. Indeed, a previous study has demonstrated a rise in uNGAL immediately after a marathon, suggesting early tubular dysfunction, although these biomarkers returned to baseline within 24 h (McCullough et al., 2011). More recently, Machado et al. (2018) have found an isolated increase in urinary NGAL in athletes who regularly practiced endurance cycling, suggesting a metabolic adaptation to exercise, rather than any pathological kidney damage.

This trial has several limitations. The main limitation is the participation rate in the study (one third); we cannot exclude that characteristics of participants (and then outcome results) differ from characteristics of non-participants, inducing a selection bias that limits generalization of our results. The sophisticated collection of serum and urinary biochemical data in such wilderness environment of the race was both strength and limitation of this study. The severity of methods, especially exclusion criteria concerning the use of NSAIDs, led to a very small sample of finishers with complete dataset, and may not have been fully representative of the prevalence of disease. It is also important to emphasize that in present study the ambient temperature was low during the race (8.6–11.1°C, rainy day) and runners had constant access to water, and the risk of volume depletion induced by exercise was low. Combined to exclusion of use of NSAIDs, our results would provide valid insight on the strict impact of running an ultramarathon on kidney function. However, future studies need to be conducted in runners that might be at an increased risk of AKI, i.e., those that were dehydrated and those taking NSAIDs to compare the essential prevalence of AKI in these groups vs. controls.

## Conclusion

We did not observe a significant decrease in glomerular filtration rate following an ultramarathon race of 120 km in tempered conditions and in subjects with strictly no use of NSAIDs during the event. The prevalence of acute kidney injury was found from 0 to 4.2% depending on the methodology applied to calculate GFR. However, we demonstrated an increase in levels of NGAL that may indicate kidney injury, leading to an elevated prevalence of AKI to 12.5% in probable properly hydrated runners. Whether this former methodology leads to the overdiagnosis of AKI and simply reflects metabolic adaptation induced by strenuous exercise or indicates deleterious structural tubular damage as pathological kidney damage, needs to be further clarified. However, our results demonstrated a very low prevalence of AKI and no evidence that ultra-endurance running can cause important kidney damage in properly hydrated subjects with no use of NSAIDs.

## Data Availability Statement

The datasets generated for this study are available on request to the corresponding author.

## Ethics Statement

The studies involving human participants were reviewed and approved by Comité de Protection des Personnes Nord-Ouest IV-n° 2017-A00808-45. The patients/participants provided their written informed consent to participate in this study.

## Author Contributions

MP, CT, J-CV, and BC contributed the conception and design of the study. CT, MP, NT, and HR organized the database. NT and HR performed the statistical analysis. BC wrote the first draft of the manuscript. MP, CT, LF, EA, OH, NT, HR, J-VC, and BC wrote the sections of the manuscript. All authors contributed to manuscript revision, read, and approved the submitted version.

### Conflict of Interest

The authors declare that the research was conducted in the absence of any commercial or financial relationships that could be construed as a potential conflict of interest.
